# Regulation of cell death receptor S-nitrosylation and apoptotic signaling by Sorafenib in hepatoblastoma cells^☆^

**DOI:** 10.1016/j.redox.2015.07.010

**Published:** 2015-07-22

**Authors:** A. Rodríguez-Hernández, E. Navarro-Villarán, R. González, S. Pereira, L.B. Soriano-De Castro, A. Sarrias-Giménez, L. Barrera-Pulido, J.M. Álamo-Martínez, A. Serrablo-Requejo, G. Blanco-Fernández, A. Nogales-Muñoz, A. Gila-Bohórquez, D. Pacheco, M.A. Torres-Nieto, J. Serrano-Díaz-Canedo, G. Suárez-Artacho, C. Bernal-Bellido, L.M. Marín-Gómez, J.A. Barcena, M.A. Gómez-Bravo, C.A. Padilla, F.J. Padillo, J. Muntané

**Affiliations:** a Institute of Biomedicine of Seville (IBiS), Hospital Universitario “Virgen del Rocío”/CSIC/Universidad de Sevilla, Av. Manuel Siurot s/n, 41013 Sevilla, Spain; b Departament of Biochemistry and Molecular Biology, University of Cordoba, Instituto Maimónides de Investigación Biomédica de Córdoba (IMIBIC), 14071 Córdoba, Spain; c Department of General Surgery, Hospital Universitario “Virgen del Rocío” – “Virgen Macarena”/Instituto de Biomedicina de Sevilla (IBiS)/CSIC/Universidad de Sevilla, Sevilla, Spain; d Hepato-Biliary Surgery Unit, Hospital Universitario “Miguel Servet”, Zaragoza, Spain; e Hepato-Biliary-Pancreatic and Liver Transplant Service, Hospital Universitario “Infanta Cristina”, Badajoz, Spain; f Department of General Surgery and Department of Pathology, Hospital Universitario “Rio Hortega”, Valladolid, Spain; g Department of Pathology, Hospital Universitario “Rio Hortega”, Valladolid, Spain; h CENTRO DE INVESTIGACIÓN BIOMÉDICA EN RED de Enfermedades Hepáticas y Digestivas (CIBERehd), Spain

**Keywords:** DAF-FM, 4-amino-5-methylamino-2′,7′-difluorofluorescein diacetate, NONOate, 1,1-diethyl-2-hydroxy-2-nitroso-hydrazine sodium, FADD, Fas-associated death domain, H_2_O_2_, hydrogen peroxide, NO, nitric oxide, NOS, nitric oxide synthase, CSNO, S-nitroso-l-cysteine, O_2_^·−^, superoxide anion, TNF-R1, tumor necrosis factor receptor type I, TRAIL-R1, tumor necrosis factor-related apoptosis-inducing ligand type I, Sorafenib, Hepatoblastoma, S-nitrosylation, NO, Apoptosis, Death-receptors

## Abstract

Nitric oxide (NO) plays a relevant role during cell death regulation in tumor cells. The overexpression of nitric oxide synthase type III (NOS-3) induces oxidative and nitrosative stress, p53 and cell death receptor expression and apoptosis in hepatoblastoma cells. S-nitrosylation of cell death receptor modulates apoptosis. Sorafenib is the unique recommended molecular-targeted drug for the treatment of patients with advanced hepatocellular carcinoma. The present study was addressed to elucidate the potential role of NO during Sorafenib-induced cell death in HepG2 cells. We determined the intra- and extracellular NO concentration, cell death receptor expression and their S-nitrosylation modifications, and apoptotic signaling in Sorafenib-treated HepG2 cells. The effect of NO donors on above parameters has also been determined. Sorafenib induced apoptosis in HepG2 cells. However, low concentration of the drug (10 nM) increased cell death receptor expression, as well as caspase-8 and -9 activation, but without activation of downstream apoptotic markers. In contrast, Sorafenib (10 µM) reduced upstream apoptotic parameters but increased caspase-3 activation and DNA fragmentation in HepG2 cells. The shift of cell death signaling pathway was associated with a reduction of S-nitrosylation of cell death receptors in Sorafenib-treated cells. The administration of NO donors increased S-nitrosylation of cell death receptors and overall induction of cell death markers in control and Sorafenib-treated cells. In conclusion, Sorafenib induced alteration of cell death receptor S-nitrosylation status which may have a relevant repercussion on cell death signaling in hepatoblastoma cells.

## Highlights

• Sorafenib induces apoptosis in hepatoblastoma cells.
• Low concentration of Sorafenib (10 nM) increases the expression of cell death receptor, as well as caspase-8 and -9 activation.
• High concentration of Sorafenib (10 µM) reduced cell death receptor S-nitrosylation that promoted a shift to more potent activation of downstream apoptotic markers.

## Graphical abstract

**Figure:**
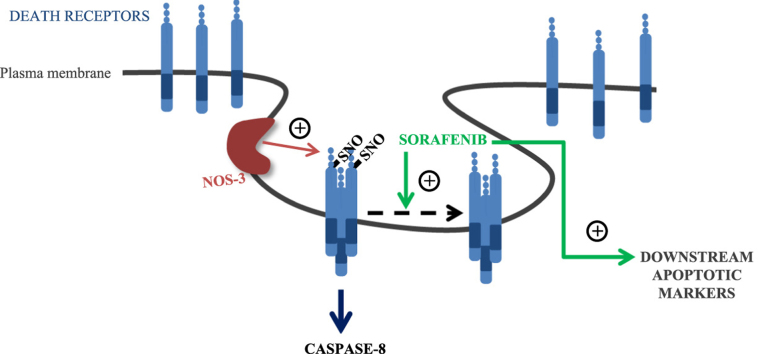

## 1 Introduction

Nitric oxide (NO) is a lipophilic, highly diffusible, and short-lived physiological messenger [1]. NO is synthesized by three different gene-encoded NO synthases (NOS) in mammals: neuronal NOS (nNOS or NOS-1), inducible NOS (iNOS or NOS-2) and endothelial NOS (eNOS or NOS-3). The expression of NOS-2 is induced by inflammatory stimuli, while NOS-1 and NOS-3 are constitutively expressed [2]. NO regulates a variety of important physiological responses, including vasodilation, respiration, cell migration, immune response and apoptosis. Different clinical trials have demonstrated that the infusion of NO donors increases the effectiveness of chemotherapy and radiotherapy in patients with cancer [3,4]. NO appears to induce genotoxic lesions, and promotes angiogenesis, tumor cell growth, and invasion [5]. However, NO is able to exert antitumoral properties, as well as increase susceptibility to chemotherapy in different experimental *in vivo* and *in vitro* models [6].

The key hallmarks of cancer cells are unlimited replicative potential, insensitivity to growth-inhibitory signals, evasion of apoptosis, cellular stress, and sustained angiogenesis, invasiveness and metastatic potential [7]. The extension of several physiopathological mechanisms involved in cell proliferation, and homeostasis is limited by the co-activation of the cell death process [8]. The expression of proteins that promote cell proliferation and tumor progression requires the expression of antiapoptotic proteins or the inactivation of essential proapoptotic proteins in order to progress [9]. This assumption is confirmed by the finding that deregulated proliferation alone is not sufficient for tumor formation. The acquisition of resistance of tumor cells to apoptosis is an essential feature of cancer development. Cell death receptors, such as the tumor necrosis factor receptor type I (TNF-R1, p55, DR1), Fas/APO-1 (CD95, DR2), and tumor necrosis factor-related apoptosis-inducing ligand type I (TRAIL-R1, DR4) and type II (TRAIL-R2, DR5), are members of the tumor necrosis factor receptor (TNF-R) family. All members within the family are characterized by the presence of a cysteine-rich extracellular domain, which defines their ligand specificity [10,11], and a cytoplasmic death domain of around 80 amino acids, which plays a central role in the activation of the caspase-dependent pathway and induction of apoptosis [12,13].

We [14] and others [15] have shown that NO sensitizes tumor cells by increasing cell death receptor expression on cancer cells. The post-translation modifications of the cell death receptors might promote or prevent its redistribution into lipid rafts and consequently, their susceptibility to cell death. In particular, pro-apoptotic stimuli, such as CD95L, induce an epidermal growth factor receptor (EGFR)-catalyzed tyrosine phosphorylation of CD95-death receptor in hepatocytes, as a prerequisite for CD95-translocation to the plasma membrane, formation of the DISC and execution of apoptotic cell death [16]. In contrast, CD95 tyrosine nitration by peroxinitrite prevents its phosphorylation and cell death in Huh 7 cells [17]. NO donor or NOS-2 overexpression induces S-nitrosylation of Cys199 and Cys304, located in the cytoplasmic domain of CD95, increasing its migration to lipid raft and apoptosis in colon and breast cancer cells [18].

The administration of antitumoral agents, such as doxorubicin, cisplatin, adriamycin and bleomycin increases the expression of cell death receptors and/or their ligands, as well as other components of the cell death pathways such as Fas-associated death domain (FADD), pro-caspase-8, pro-caspase-3, the long isoform of pro-caspase-2 and Bax in different carcinoma cell lines [19–23]. Sorafenib, a multi-kinase inhibitor which inhibits proliferation and angiogenesis, is the recommended treatment for patients with locally advanced/metastatic hepatocarcinoma [24,25]. The increased susceptibility to cell death by Sorafenib is associated with down-regulation of cell survival pathways in hepatoma cells [26,27]. However, discrepancies exist regarding the regulation of extrinsic cell death pathways by Sorafenib in different tumor cell lines [28,29]. In addition, Sorafenib has been shown to dose-dependently induce oxidative stress, such as superoxide anion (O_2_^·−^), hydrogen peroxide (H_2_O_2_) and NO, in HepG2 cells [30]. The aim of the present study was to determine the capacity of Sorafenib to regulate the expression of cell death receptor and/or its S-nitrosylations, as well as extrinsic apoptotic signaling in hepatoblastoma cells. Data showed that the drug reduces S-nitrosylation of cell death receptors which was related to a shift from moderate extrinsic cell death pathway to drastic increase of downstream apoptotic markers in HepG2 cells.

## 2 Material and methods

### 2.1 Materials

All reagents were from Sigma-Aldrich Chemical Co. (St Louis, Missouri, USA) unless otherwise stated. 4-Amino-5-methylamino-2′,7′-difluorofluorescein diacetate (DAF-FM) was obtained from Molecular Probes (Cat. D-23842). S-nitroso-l-cysteine (CSNO) was prepared according to the procedure described elsewhere [31] by incubation of l-cysteine with acidified sodium nitrite and quantification by absorbance at 334 nm using a molar absorption coefficient of 0.74 mM^−1^ cm^−1^. Sorafenib Tosylate was purchased from Carbosynth (Carbosynth Ltd., Berkshire, United Kingdom).The study protocol has been approved by the Ethical Committee of the Institution.

### 2.2 Cell lines and culture conditions

HepG2 cell line was obtained from the ATCC-LGC (Barcelona, Spain). Cells were negative for mycoplasma contamination. Cells were routinely maintained in EMEM (Eagle´s Minimum Essential Medium) (Sigma-Aldrich Chemical Co) pH 7.4 supplemented with 10% fetal bovine serum (F7524, Sigma-Aldrich, Lot No: 022M3395, endotoxin <0.2 EU/ml), 2.2 g/l HCO_3_Na, 1 mM sodium pyruvate, 100 U/ml penicillin, 100 µg/ml streptomycin, 0.25 µg/ml amphotericin and the corresponding selection antibiotic in 5% CO_2_ in air at 37 °C.

Cells were maintained for 24 h before administration of Sorafenib (0, 10 nM and 10 µM). 1,1-Diethyl-2-hydroxy-2-nitroso-hydrazine sodium (NONOate) (5 µM) or CSNO (5 µM) was administered 30 min before Sorafenib, and maintained for 48 h. The parameters were assessed at 12, 24 and 48 h after Sorafenib administration. Culture medium was replaced every 24 h.

### 2.3 Measurement of cell death

Caspase-8, Caspase-9 and Caspase-3-associated activity were determined using Caspase-Glo® Assay Systems (G8201, G8211 and G8091, Promega, Madison, Wisconsin, USA). Cells were treated with Caspase-Glo® Reagent in an “add-mix-measure” format resulting in cell lysis, caspase-8-dependent cleavage of the substrate and generation of a “glow-type” luminescent signal. The signal generated is proportional to the amount of caspase activity. The values are extrapolated into a calibration curve included in the assay.

HepG2-fixed cells cultured in glass-Petri dish were used for measuring apoptosis by TUNEL assay (Promega, G3250). The assay measures the fragmented DNA of apoptotic cells by catalytically incorporating fluorescein-12-dUTP at 3′-OH DNA ends using the enzyme Terminal Deoxynucleotidyl Transferase (TdT), which forms a polymeric tail using the principle of the TUNEL assay. The fluorescein-12-dUTP-labeled DNA was visualized using an Olympus BX61 microscope. Fluorescence quantification was performed using Leica Application Suite Advanced Fluorescence software and ImageJ software.

### 2.4 Expression of TNF-R1, CD95 and TRAIL-R1

Cells were washed twice with PBS (with Ca^2+^) and treated with lysis buffer (50 mM HEPES pH. 7.5, 150 mM NaCl, 5 mM EDTA, 1% NP-40) including 1 mM PMSF, 1 mM NaF, 1 mM Na_3_VO_4_ and a commercial protease inhibitor cocktail containing AEBSF-HCl (Serine protease), Aprotinin (Serine protease), Bestatin (Aminopeptidase B and Leucine Aminopeptidase), E-64 (Cysteine protease), Leupeptine (Cysteine protease and trypsin-like serine proteases), Pepstatin A (Aspartic proteases) (P8340, Sigma-Aldrich Chemical Co.). Culture medium was collected and centrifuged for 5 min 4 °C at 400 g, and detached cells transferred to cell lysate, and maintained for 20 min on ice with vortex each 5 min. After centrifugation at 15,520 g for 5 min at 4 °C, the supernatant was stored at −20 °C for the measurement of NO-end products. The expression of TNF-R1, CD95 and TRAIL-R1 protein expression were determined by SDS-PAGE coupled to Western-blot analysis. Proteins (50–100 μg) were separated by 10–12% SDS-PAGE and transferred to PVDF membranes. The membranes were incubated with the corresponding commercial primary antibodies against TNF-R1 (1/250) (sc-7895, Santa Cruz Biotechnology Inc.), CD95 (1/250) (sc-715, Santa Cruz Biotechnology Inc.; Delaware, California, USA), and TRAIL-R1 (1/250) (sc-6823, Santa Cruz Biotechnology Inc.). The corresponding anti-rabbit (1/10000) (sc-2301, Santa Cruz Biotechnology, Inc.), anti-mouse (1/10000) (sc-2031, Santa Cruz Biotechnology Inc.,) or anti-goat (1/10000) (sc-, Santa Cruz Biotechnology, Inc.) secondary antibodies were coupled to horseradish peroxidase revealing protein content by Pierce ECL Western Blotting Substrate (Thermo Scientific, Waltham, Massachusetts, USA). G3PDH (0.05 μg/ml) (G8795, Sigma) were used as cell protein loading control in cell membrane embedded proteins.

### 2.5 Measurement of NO

The production of NO was measured by quantification of its related end products, nitrite/nitrate in culture medium obtained as described above. In the assay, nitrate was converted to nitrite by nitrate reductase (EC 1.6.6.2) and total nitrite was measured using the Griess reaction [32]. Briefly, the samples were incubated with nitrate reductase (0.2 U/ml), FAD (5 mM) and NADPH (50 mM) for 20 min at 37 °C. The reaction was stopped by the addition of sodium pyruvate (10 mM) and lactate dehydrogenase (24 mg/ml) for 5 min at 37 °C, and precipitated with 1.4% ZnSO_4_. Total nitrite reacted with Griess reagent (1% sulfanilamide, 2.5% PO_4_H_3_, 0.1% n-naphthyl-ethylene-diamine) for 10 min at 37 °C, and was read at 540 nm using a Infinite 200 PRO Microplate Reader (TECAN).

The *in situ* production of NO was monitored using a fluorescent probe, such as DAF-FM diacetate (D-23842, Molecular Probes Europe BV, Leiden, The Netherlands), hydrolyzed by intracellular esterases to form DAF-FM in cytoplasm, and oxidized by NO to yield the highly fluorescent Triazole form (DAF-FM-T). Stock solution of DAF-FM diacetate was maintained in DMSO and stored at −20 °C until use. Cells were incubated with DAF-FM diacetate (2.5 μM) for 30 min in culture medium. Cells were washed with PBS, in order to avoid any interference with the measurement of cell fluorescence. The production of NO was *in situ* assessed as the enhancement on the fluorescence at Ex 495 nm/Em 515 nm measured using a Infinite 200 PRO Microplate Reader (TECAN). The corresponding controls were carried out in cells incubated in the presence/absence of solvent, dye and cells.

### 2.6 Detection of S-nitrosylation in cell death receptors by the biotin switch assay

The procedure was performed as previously described [33] with recently described modifications [34]. Cells were treated with lysis solution (50 mM Tris–HCl pH 7.4, 300 mM NaCl, 5 mM EDTA, 0.1 mM neocuproine, 1% Triton X-100, 5 µg/mL aprotinin and 10 µg/mL leupeptin), and after centrifugation at 10,000 g the supernatants (0.5 µg/µL) were incubated with 4 volumes of blocking buffer (225 mM HEPES, pH 7.7, 0.9 mM EDTA, 90 µM neocuproine, 2.5% SDS and 20 mM methyl methane thiosulfonate or MMTS) for 20 min at 50 °C, and precipitated with 3–4 volumes of cold acetone. The dried pellet was resuspended in 500 µL HENS (HEN and 1% SDS) with 100 mM sodium ascorbate and 167 µL 4 mM Biotin-HPDP for 1 h, precipitated with cold acetone, and dried pellet resuspended in 200 µL HENS buffer and 800 µL of neutralization buffer (20 mM HEPES pH 7.7, 100 mM NaCl, 1 mM EDTA, 0.5% Triton X-100). Samples were treated with 45 µL Neutravidin Plus UltralinK resin (Pierce) for 1 h in agitation, washed (20 mM HEPES pH 7.7, 600 mM NaCl, 1 mM EDTA and 0.5% Triton X-100), and incubated in 20 mM HEPES pH 7.7, 100 mM NaCl, 1 mM EDTA, and 100 mM 2-mercaptoethanol for 20 min at 37 °C. After centrifugation, supernatants were loaded into 10% SDS-PAGE electrophoresis in reducing conditions. The expression of cell death receptors was detected by Western Blot analysis as described above.

### 2.7 Statistical analysis

Results are expressed as mean±SE of three (TUNEL and biotin switch assays), six (Western-blot images) and seven (caspase activities and NO-related measurements) independent experiments. Normal distribution was assessed by Shapiro–Wilk test. Homogeneity of variances was determined by Levene test. Data were compared using the analysis of variance with the Least Significant Difference’s test as post-hoc multiple comparison analysis. The statistical differences were set at *p*≤0.05. The groups in the bar graphs showing different letters (a–e) were statistically different.

## 3 Results

### 3.1 Induction of apoptosis by Sorafenib

**Fig. 1:**
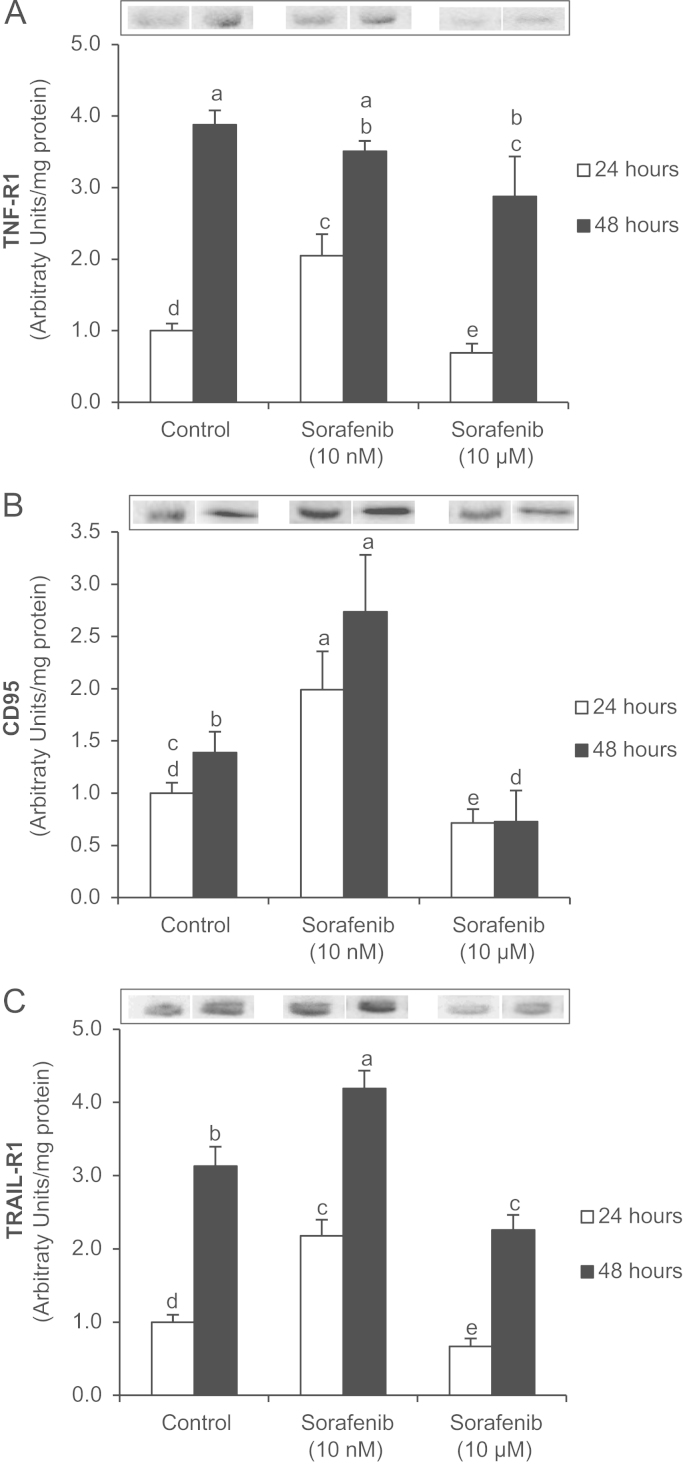
Regulation of TNF-R1 (A), CD95 (B) and TRAIL-R1 (C) expression by Sorafenib in HepG2 cells. The expression of cell death receptors has been assessed by Western-blot analysis at 24 and 48 h after Sorafenib (0, 10 nM and 10 µM) administration. GAPDH was used as internal protein loading. The statistical analysis of densitometric values of the spots is shown below the blot. Data are expressed as mean±SEM. The groups with different letters (a, b, c, d or e) were significantly different (*p*≤0.05). The images are representative of six independent experiments.

**Fig. 2:**
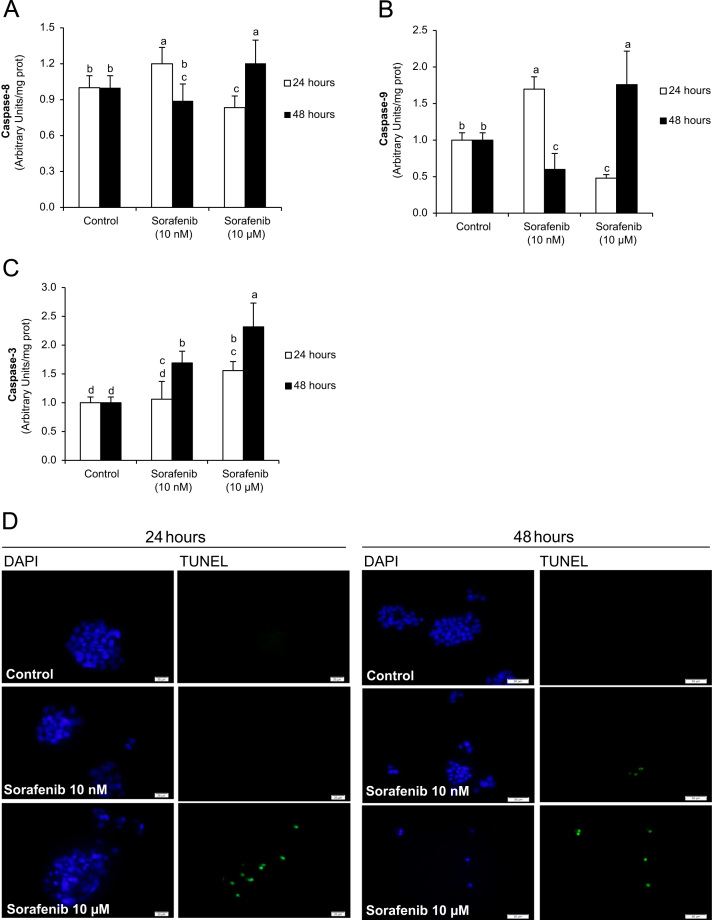
Regulation of caspase-8 (A), caspase-9 (B), caspase-3 (C) and DNA fragmentation (D) by Sorafenib in HepG2 cells. The activity of caspases was detected by commercial chemiluminescence-based assay. DNA fragmentation was determined by TUNEL assay. The variables were evaluated at 24 and 48 h after Sorafenib (0, 10 nM and 10 µM) administration. Data are expressed as mean±SEM of seven independent experiments. The groups with different letters (a, b, c or d) were significantly different (*p*≤0.05). The images obtained from TUNEL assays are representative of three independent experiments.

Sorafenib increases cell death in hepatoma cells [26]. However, discrepancies exist between the involvement of extrinsic and intrinsic cell death pathway during Sorafenib-induced cell death [28,35]. Sorafenib dose-dependently regulated apoptotic cell signaling in hepatoblastoma cells. The expression of cell death receptors (TNF-R1, CD95 and TRAIL-R1) (Fig. 1), as well as caspase-8 (Fig. 2A) and -9 (Fig. 2B) activation was induced at 24 h after administration of Sorafenib at low concentration (10 nM). However, higher concentration of Sorafenib (10 µM) reduced the above markers, and promoted the activation of downstream apoptotic markers such as caspase-3 (Fig. 2C) and TUNEL-staining (Fig. 2D) in HepG2 cells. Although the expression of cell death receptors (CD95 and TRAIL-R1) was still increased at low concentration of Sorafenib (10 nM), a significant rise of caspase-3 activity was observed that correlated with a reduction of caspase-8 and -9 at 48 h. High doses of Sorafenib (10 µM) increased both extrinsic and intrinsic pathways at 48 h in HepG2 cells.

### 3.2 Regulation of NO-generation and S-nitrosylation of cell death receptors by Sorafenib

**Fig. 3:**
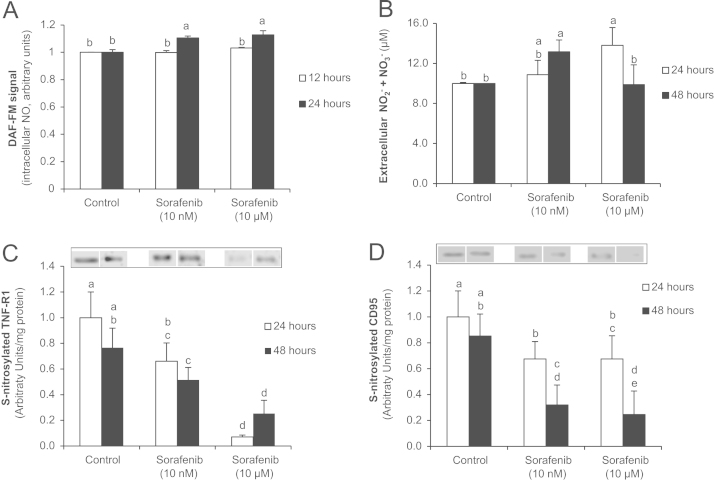
Regulation of NO generation and S-nitrosylation of TNF-R1 (C), CD95 (D) and TRAIL-R1 (E) by Sorafenib in HepG2 cells. NO intracellular (A) and extracellular (B) concentrations were determined using DAF-FM and NO-end products, respectively. S-nitrosylation was assessed by the biotin switch assay which was coupled to Western-blot analysis at 24 and 48 h after Sorafenib (0, 10 nM and 10 µM) administration. The statistical analysis of densitometric values of the spots is shown below the blot. Data are expressed as mean±SEM of seven independent experiments. The groups with different letters (a, b, c, d or e) were significantly different (*p*≤0.05). The images are representative of three independent experiments (biotin switch) and six independent experiments (NO-related measurements).

Sorafenib increased NO intracellular concentration measured by DAF-FM (Fig. 3A, 24 h) and NO-end products in culture medium (Fig. 3B, 24 h). The rise of NO-end product concentration was only observed at low Sorafenib concentration (10 nM). The medium was replaced every 24 h, so the measurement of NO-end product concentration in culture medium corresponds to its accumulation between 0–24 h and 24–48 h. This fact may explain the reduction of NO-end product concentration in culture medium from cells treated with high dose of Sorafenib (10 µM) at 48 h in which NO release appeared to be lower compared to that observed at 0–24 hours. Interestingly, this effect of the drug was related to a drastic dose-dependent reduction of S-nitrosylation in TNF-R1 (Fig. 3C) and CD95 (Fig. 3D). S-nitrosylation of TRAIL-R1 was not detected in the absence of NO donor (data not shown).

### 3.3 Role of NO-donor on cell death in control and Sorafenib-treated hepatoblastoma cells

**Fig. 4:**
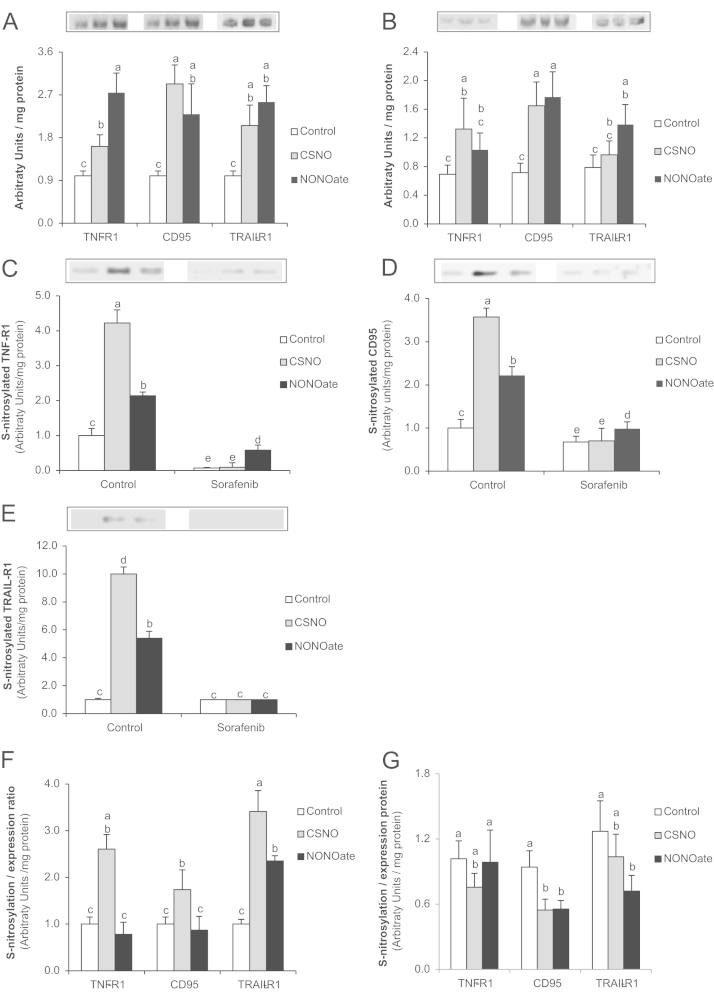
Effect of NO donors on the expression of cell death receptors in control (A) and Sorafenib (B)-treated HepG2 cells, as well as the S-nitrosylation of TNF-R1 (C), CD95 (D) and TRAIL-R1 (E) in cells. NONOate (5 µM) or CSNO (5 µM) was administered 30 min before Sorafenib (10 µM). S-nitrosylation was assessed by the biotin switch assay which was coupled to Western-blot analysis at 24 h after Sorafenib administration. The estimation of S-nitrosylation/expression ratio of cell death receptor is shown in control (F) and Sorafenib (G)-treated cells. The statistical analysis of densitometric values of the spots is shown below the blot. Data are expressed as mean±SEM. The groups with different letters (a, b, c, d or e) were significantly different (*p*≤0.05). The images are representative of three independent experiments.

**Fig. 5:**
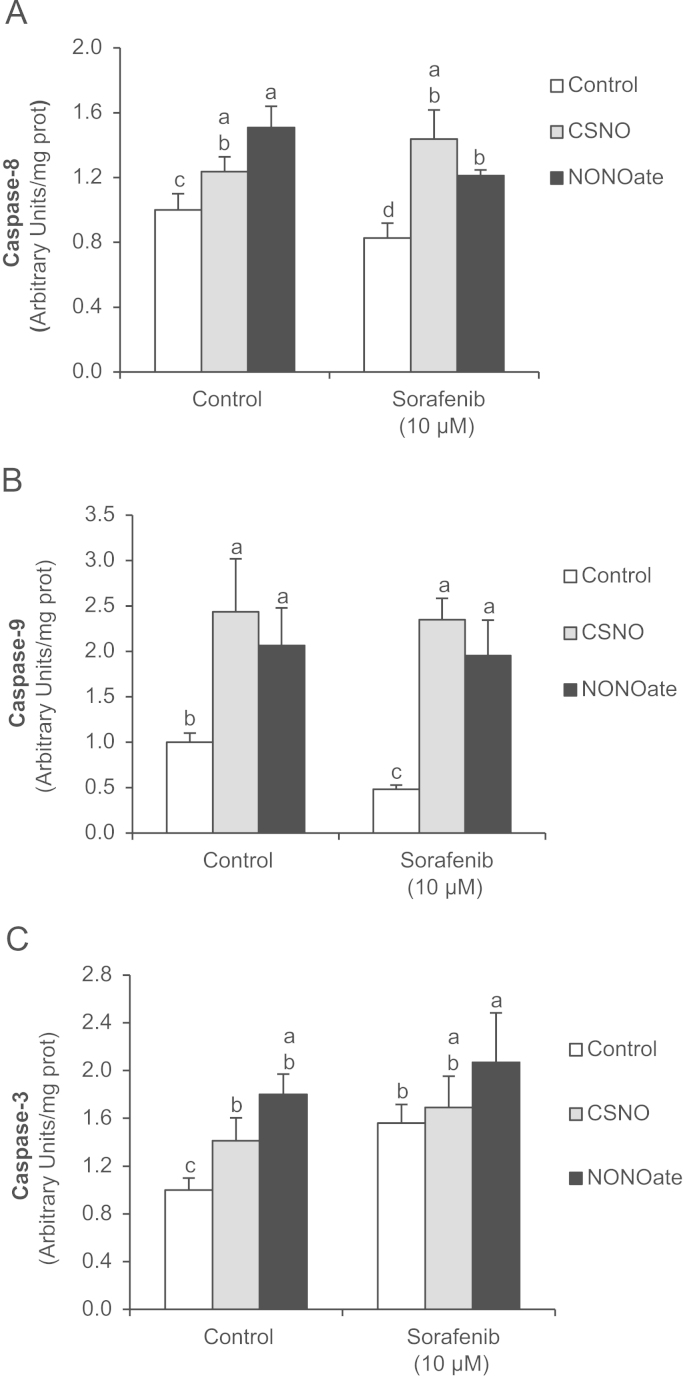
Effect of NO donors on caspase-8 (A), caspase-9 (B) and caspase-3 (C) activation by Sorafenib in HepG2 cells. CSNO (5 µM) or NONOate (5 µM) was administered 30 min before Sorafenib. The activity of caspases was detected by commercial chemiluminescence-based assay at 24 after Sorafenib (0 and 10 µM) administration. Data are expressed as mean±SEM of seven independent experiments. The groups with different letters (a, b, c or d) were significantly different (*p*≤0.05).

The administration of NO donors increased the expression of cell death receptor in control cells (Fig. 4A), as well as S-nitrosylation of TNF-R1 (Fig. 4C), CD95 (Fig. 4D) and TRAIL-R1 (4E) in control cells. The estimation of S-nitrosylation/expression ratio showed that CSNO increased S-nitrosylation of cell death receptor stronger than NONOate in control cells (Fig. 4F). The administration of Sorafenib (10 µM) changed the effect of NO donors in HepG2. In this sense, Sorafenib reduced the increase of cell death receptor expression induced by NO donors to values close to control levels (TNF-R1 and TRAIL-R1) (Fig. 4B), as well as the effect of NO donors on S-nitrosylation of cell death receptors (Fig. 4C–E) in HepG2 cells. Interestingly, the estimation of S-nitrosylation/expression ratio showed that Sorafenib reduced S-nitrosylation of CD95 and TRAIL-R1 in NO donor-treated cells (Fig. 4G). The administration of NO donors increased caspase-8 (Fig. 5A), caspase-9 (Fig. 5B) and caspase-3 (Fig. 5C) in control and Sorafenib-treated cells.

## 4 Discusion

The biological activity of NO may be through cGMP-dependent and cGMP-independent pathways which play a relevant role in pathophysiological conditions [36]. NO induces cGMP-dependent protein kinases, cyclic-nucleotide-gated ion channels and cGMP-regulated phosphodiesterases [6]. However, during the last decade cGMP-independent reactions gained considerable interest. A variety of effects are achieved through its interactions with targets via redox and additive chemistry, that may promote covalent modifications of intracellular proteins as well as oxidation events that do not require attachment of the NO group [37]. The most prominent and recognized NO reaction with thiols groups of cysteine residues is called S-nitrosylation or S-nitrosation, which leads to the formation of more stable nitrosothiols [38].

Several NO-dependent signal transduction pathways are related to protein S-nitrosylation, which in terms of cell death may exert either pro-apoptotic activity as a consequence of the alteration of mitochondrial potential and free radical generation [39], or anti-apoptotic such as GSNO- or thioredoxin-dependent S-nitrosylation of caspases [40], PKCε which reduces cFLIP release from DISC complex [41], and cFLIP avoiding its degradation through proteosomal degradation [42].

The key hallmarks of cancer cells are unlimited replicative potential, insensitivity to growth-inhibitory signals, evasion of apoptosis, cellular stress, and sustained angiogenesis, invasiveness and metastatic potential [7]. The alteration of cell death signaling is frequently observed in cancer [43–45]. The role of NO in tumor progression depends on the activity and localization of NOS isoforms, concentration and duration of NO exposure, cellular sensitivity, and hypoxia/re-oxygenation status [36]. The induction of nitrosative stress increases CD95 expression and cell death in pulmonary artery smooth muscle cells [46] and neurons [47]. We have recently shown that p53 mediates the increase of cell death receptor expression and cell death induced by the exogenous administration of NO donor and/or NOS-3 overexpression in differentiated hepatoma cells lines [14]. In addition, S-nitrosylation of critical cysteine residues of CD95 modulates CD95 membrane trafficking and cell death stimulation in hepatoma cancer cells [18]. The induction of caspase-8 activation was also related to S-nitrosylation of TRAIL-R1 in melanoma (A375), renal carcinoma (ACHN), and ovarian carcinoma (NIH-OVCAR-3) cells treated with nitrosylcobalamin [48].

Sorafenib, a multi-kinase inhibitor which inhibits proliferation and angiogenesis, is the recommended treatment for patients with locally advanced/metastatic hepatocarcinoma [24,25]. Sorafenib exerts antitumoral activity targeting tyrosine kinase proliferating receptor, and on Raf-1 and B-Raf thus inhibiting RAF/MEK/ERK signaling pathway [49]. Although, Sorafenib increases apoptosis several discrepancies exist regarding the involvement of extrinsic or intrinsic pathways by Sorafenib in different tumor cell lines [28,29].

The present study demonstrated that low concentration of Sorafenib (10 nM) was able to increase the expression of cell death receptors (TNF-R1, CD95 and TRAIL-R1) that correlated to high caspase-8 and -9 activities but without significant increase of downstream apoptotic signaling such as caspase-3 activity and DNA fragmentation (Figs. 1 and 2). Interestingly, high Sorafenib concentration (10 µM) reduced basal level of cell death receptor expression and caspase-8 and -9 activation, but increased all those related to downstream apoptotic signaling at 24 h after administration. Caspase-8 and -9 activities relapse at 48 h in cells treated with high Sorafenib concentration. The present study and other [30] have shown an increased NO production induced by Sorafenib in hepatoma cells. The administration of Sorafenib increased the intracellular NO concentration (DAF-FM) and in culture medium (NO-end products) that were related to reduction of S-nitrosylation of cell death receptors (TNF-R1 and CD95) (Fig. 3). This effect was particularly evident in cells treated with high Sorafenib concentration (10 µM) at 24 h. In these conditions, the expression of cell death receptors, as well as caspase-8 and -9 activities were reduced compared to control level (Figs. 1 and 2). It were interesting to observe that the recovery of TNF-R1 and TRAIL-R1 expression, as well as caspase-8 and -9 in cells treated with Sorafenib (10 µM) (Figs. 1 and 2) were related to a recovery of S-nitrosylation of TNF-R1 (Fig. 3) at 48 h. Our data suggest that Sorafenib might transitorily activate denitrosylating-like activity. Thioredoxin-1 has been shown to actively denitrosylated cytosolic caspase-3 and thereby maintains a low steady-state amount of S-nitrosylation [50]. CSNO treatment also increased S-nitrosoglutathione reductase (GSNOR) activity that reduces SNO content in human hepatocytes [51].

The administration of low concentrations of NO donor such as CSNO and NONOate (5 µM) induced a permissive induction of cell death (up to 48 h). This experimental approach allows us to combine NO donor and Sorafenib treatments. NO donors drastically induced S-nitrosylation of cell death receptors and increased caspase-8, caspase-9 and caspase-3 activities in control cells. The estimation of S-nitrosylation/expression ratio showed that CSNO was more effective than NONOate inducing S-nitrosylation of cell death receptor (Fig. 4E). This could be due to the characteristic of CSNO molecule, which is a permeable nitrosothiol whose effect in the induction of S-nitrosylation is more efficient than NONOate. However, this difference was not accompanied with a reduction of caspase-8 and -9 activation by NONOate compared to CSNO treatment. Sorafenib (10 µM) reduced the expression of cell death receptors and their S-nitrosylation of cell death receptors (TNF-R1 and CD95) compared to the level observed in control and NO donor-treated HepG2 cells (Figs. 1, 3, 4B–E). Interestingly, Sorafenib (10 µM) reduced the estimated value of S-nitrosylation/expression ratio in NO donor-treated cells. S-nitrosylation of cell death receptors seems to be relevant for their trafficking to lipid raft and induction of apoptosis in hepatocytes [18]. In concordance, the reduction of cell death receptor S-nitrosylation by Sorafenib was related to a reduction of caspase-8 and -9, but a relevant increase of caspase-3 in HepG2 cells (24 h). The presence of NO generation system in cell membrane may be relevant for cell signaling. In this sense, the antitumoral properties of NOS-3 observed by our group [14,52] may be reinforced by its localization in lipid raft domains or caveolae fraction [53]. In this sense, the extrinsic cell death signaling has been shown to be regulated by caveolin-1, a specific protein of lipid raft compartment, in physiopathological cellular conditions [54–56].

The present study suggests that the down-regulation of S-nitrosylation of cell death receptors by Sorafenib may be critical to reinforce its downstream pro-apoptotic properties of the drug overwhelming upstream survival pathways in hepatoma cells. More studies will allow the identification of potential denitrosylating activity induced by Sorafenib which may affect the trafficking or activity of cell death receptors into lipid raft in hepatoblastoma cells.
