# Corrigendum to ‘Regulation of cell death receptor S-nitrosylation and apoptotic signaling by Sorafenib in hepatoblastoma cells’[Redox Biol 6(2015):174–182]

**DOI:** 10.1016/j.redox.2023.102744

**Published:** 2023-05-26

**Authors:** A. Rodríguez-Hernández, E. Navarro-Villarán, R. González, S. Pereira, L.B. Soriano-De Castro, A. Sarrias-Giménez, L. Barrera-Pulido, J.M. Álamo-Martínez, A. Serrablo-Requejo, G. Blanco-Fernández, A. Nogales-Muñoz, A. Gila-Bohórquez, D. Pacheco, M.A. Torres-Nieto, J. Serrano-Díaz-Canedo, G. Suárez-Artacho, C. Bernal-Bellido, L.M. Marín-Gómez, J.A. Barcena, M.A. Gómez-Bravo, C.A. Padilla, F.J. Padillo, J. Muntané

**Affiliations:** a Institute of Biomedicine of Seville (IBiS), Hospital Universitario “Virgen del Rocío”/CSIC/Universidad de Sevilla, Av. Manuel Siurot s/n, 41013, Sevilla, Spain; b Department of Biochemistry and Molecular Biology, University of Cordoba, Instituto Maimónides de Investigación Biomédica de Córdoba (IMIBIC), 14071, Córdoba, Spain; c Department of General Surgery, Hospital Universitario “Virgen del Rocío”-“Virgen Macarena”/Instituto de Biomedicina de Sevilla (IBiS)/CSIC/Universidad de Sevilla, Sevilla, Spain; d Hepato-Biliary Surgery Unit, Hospital Universitario “Miguel Servet”, Zaragoza, Spain; e Hepato-Biliary-Pancreatic and Liver Transplant Service, Hospital Universitario “Infanta Cristina”, Badajoz, Spain; f Department of General Surgery and Department of Pathology, Hospital Universitario “Rio Hortega”, Valladolid, Spain; g Department of Pathology, Hospital Universitario “Rio Hortega”, Valladolid, Spain; h CENTRO DE INVESTIGACIÓN BIOMÉDICA EN RED de Enfermedades Hepáticas y Digestivas (CIBERehd), Spain

The authors regret < The authors regret Fig. 4A of the original manuscript was incorrect. I would appreciate if you would accept to replace by the corrected Fig. 4A.

**Figure:**
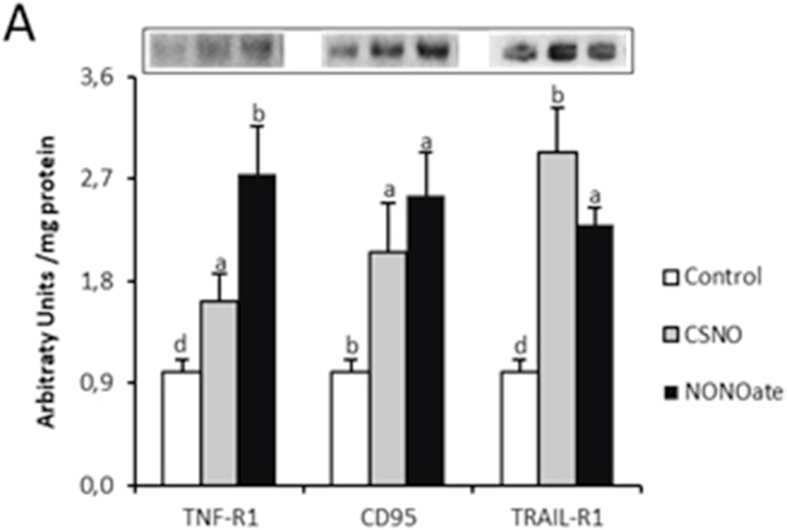

The authors would like to apologise for any inconvenience caused.

